# Effect of Resveratrol on Pregnancy, Prenatal Complications and Pregnancy-Associated Structure Alterations

**DOI:** 10.3390/antiox12020341

**Published:** 2023-01-31

**Authors:** Iman Ramli, Anna Maria Posadino, Roberta Giordo, Grazia Fenu, Manal Fardoun, Rabah Iratni, Ali H. Eid, Hatem Zayed, Gianfranco Pintus

**Affiliations:** 1 Departement de Biologie Animale, Université des Frères Mentouri Constantine 1, Constantine 25000, Algeria; 2 Department of Biomedical Sciences, University of Sassari, 07100 Sassari, Italy; 3 College of Medicine, Mohammed Bin Rashid University of Medicine and Health Sciences, Dubai 505055, United Arab Emirates; 4 Department of Pharmacology and Toxicology, American University of Beirut, Beirut 11-0236, Lebanon; 5 Department of Biology, United Arab Emirates University, Al-Ain 15551, United Arab Emirates; 6 Department of Basic Medical Sciences, College of Medicine, QU Health, Qatar University, Doha 2713, Qatar; 7 Department of Biomedical Sciences, College of Health Sciences, QU Health, Qatar University, Doha 2713, Qatar

**Keywords:** resveratrol, pregnancy, prenatal, preterm, fetus, pre-eclampsia, placenta, gestational diabetes

## Abstract

Adverse pregnancy outcomes are considered significant health risks for pregnant women and their offspring during pregnancy and throughout their lifespan. These outcomes lead to a perturbated in-utero environment that impacts critical phases of the fetus’s life and correlates to an increased risk of chronic pathological conditions, such as diabetes, obesity, and cardiovascular diseases, in both the mother’s and adult offspring’s life. The dietary intake of naturally occurring antioxidants promotes health benefits and disease prevention. In this regard, maternal dietary intake of polyphenolic antioxidants is linked to a reduced risk of maternal obesity and cardio-metabolic disorders, positively affecting both the fetus and offspring. In this work, we will gather and critically appraise the current literature highlighting the effect/s of the naturally occurring polyphenol antioxidant resveratrol on oxidative stress, inflammation, and other molecular and physiological phenomena associated with pregnancy and pregnancy conditions, such as gestational diabetes, preeclampsia, and preterm labor. The resveratrol impact on prenatal complications and pregnancy-associated structures, such as the fetus and placenta, will also be discussed. Finally, we will draw conclusions from the current knowledge and provide future perspectives on potentially exploiting resveratrol as a therapeutic tool in pregnancy-associated conditions.

## 1. Introduction

Adverse pregnancy outcomes are considered major health risks for pregnant women and their offspring during pregnancy and throughout their lifespan, with several disorders recognized, including gestational diabetes, preterm birth, fetal-growth restriction, and hypertensive disorders of pregnancy such as gestational hypertension, pre-eclampsia, and related conditions [1,2]. These outcomes lead to a perturbated in-utero environment that impacts critical phases of fetus development. An increased risk of chronic non-communicable metabolic diseases, such as cardiovascular disease, diabetes, and obesity, is often related to detrimental structural changes of major functional systems during fetal life and persists through the adult offspring’s life [3].

Dietary intake of naturally occurring antioxidants has been linked to disease prevention and health benefits [4,5,6,7]. Indeed, natural products, such as curcumin, resveratrol, and epigallocatechin-3-gallate, have been shown to exhibit a plethora of biological functions that eventually provide prevention or protection against several pathological conditions, including cancer, diabetes, obesity, cardiovascular, and neurodegenerative [8,9,10,11,12]. Among the above-mentioned natural phytonutrients, resveratrol (3,5,4′-trihydroxy-trans-stilbene), an antioxidant polyphenolic compound found in various plants and foods including grapes, berries, peanuts, apples, cacao, and soybeans, has been proven to possess a wide spectrum of biological properties including antioxidant, anti-inflammatory, neuroprotective, cardioprotective, immunomodulatory, antiplatelet, and anticancer [13,14,15,16,17,18]. In this regard, a great body of literature evidence also suggested that maternal dietary intake of antioxidants polyphenols is linked to reduced risk of maternal obesity [19] and cardio-metabolic disorders [20], positively impacting both the fetus and offspring.

This literature review will gather and discuss the current experimental evidence highlighting the effect/s of resveratrol on inflammation, oxidative stress, and other molecular and physiological phenomena associated with pregnancy and pregnancy-associated complications, including preeclampsia, gestational diabetes, and preterm labor. The effect of resveratrol on prenatal issues and pregnancy-associated structures such as the placenta and fetus will also be discussed. Finally, conclusions on the current knowledge and future directions on potentially exploiting resveratrol as a therapeutic tool in pregnancy-associated conditions are provided to the readers.

## 2. Bioavailability, Toxicity, and Hormetic Effect of Resveratrol

Due to its numerous pharmacological activities, resveratrol’s effects on humans are widely studied. Nonetheless, results from the available human clinical trials are so far contradictory concerning the protective effects of resveratrol against diseases and their sequelae [3]. Similarly, resveratrol intake effects during human pregnancy and the interpretation of its supplementation’s effectiveness are poorly understood due to variations in species, differences in the characteristics of the enrolled patients, dosage, administration routes, duration of resveratrol, supplementation and in some cases, pregnancy models complication [3]. Although resveratrol’s high absorbance and rapid metabolization without pronounced toxicity characterized its beneficial effects in several pregnant women studies [21,22,23], few reports raised doubts about possible toxicity and adverse effects following its consumption. Hence, the adequate ranges of dosage capable of inducing remarkable long-term health benefits without raising toxicity issues remain controversial [3]. Indeed, resveratrol dosage ranges differently in vitro experimental models (micromolar range in cell culture media) and in vivo experimental models (nanomolar range in the blood); hence, it is difficult to precise the optimal concentrations for a maximum beneficial effect of resveratrol in human subjects. However, concerning this aspect, studies in vivo indicate that resveratrol can reach concentration levels similar to those found to exert biological effects in vitro. Indeed, resveratrol can accumulate at relatively high concentrations (up to 32 µM) in plasma [24,25], while concentrations of resveratrol around (~10–30 µM) have been detected in other tissues like the heart, liver, and kidney [26,27]. Such a resveratrol characteristic may be of particular relevance, given its ability to cross the placental barrier and directly interact with the fetus [28]. Another challenging issue that may affect resveratrol therapeutic effects making their interpretation difficult, is its poor oral bioavailability [8]. Resveratrol’s rapid elimination from the body complicates the maintenance of the bloodstream’s acceptable therapeutic levels [29]; moreover, resveratrol is metabolized to phenolic metabolites in the liver and intestinal epithelial cells [30,31,32], and these phase II metabolites can be transformed into the aglycone forming a systemic reservoir of biologically active molecules [8]. In this light, improving resveratrol oral bioavailability by establishing new formulations that are more bioavailable and less degradable with clear pharmacokinetic routes is a crucial future research goal [33].

In human clinical trials, resveratrol was proven to be generally tolerated, yet some adverse effects, including nephrotoxicity and gastrointestinal problems, were reported [34,35]. For instance, a resveratrol dose of 450 mg/day was shown to be safe for a 60 kg individual [36]. In contrast, doses of 1000 mg/day or higher were reported to inhibit cytochrome P450 isoenzymes such as CYP3A4, CYP2C9, and CYP2D6, while activating CYP1A2 leading to interference with the pharmacokinetics of concomitantly administered drugs [37]. Moreover, adverse effects linked to resveratrol intake have been reported on different parameters, including the metabolic status of type 2 diabetes patients and the endothelial health, inflammation, and cardiovascular markers of human patients [38]. Concerning this specific topic, a comprehensive analysis of resveratrol’s adverse effects has been reported elsewhere [8]. On pregnancy, a study conducted on a Japanese macaque experimental model indicated that pregnancy resveratrol intake might cause abnormalities in the fetus, an adverse effect clearly associated with high resveratrol supplementation. The findings of this study showed that resveratrol intake (0.2, 0.5, and 1.0 mg in 5 mL administered via intravenous infusion) managed to reduce the maternal weight, improve glucose tolerance, increase uterine artery volume blood flow, and decrease placental inflammation and liver triglyceride deposition. However, resveratrol intake parallelly affected the fetal pancreatic mass (enlarged by 42%), demonstrating that pregnancy resveratrol supplementation delivers beneficial effects on maternal, placental phenotype, and fetal liver but also an unexplained and concerning alteration of the fetal pancreatic development [39]. In this regard, Cottart et al. [21] highlighted that, despite the extensive amount of data concerning the benefits derived from resveratrol intake, there is scarce research aimed at assessing its harmful effects, and human clinical studies in this regard are limited [21].

Both in vitro and in vivo resveratrol’s biological effects are associated with its hermetic behavior, where low resveratrol doses induce beneficial effects while higher doses usually have a harmful effect [40]. Evidence suggests that such a phenomenon is linked to the biphasic resveratrol effect on the cellular redox state, antioxidant at low doses and pro-oxidant at high ones [15,40,41]. As such, many of the controversial results in the literature might be due to this hormetic aspect, besides other elements related to enrolled patients, doses, and duration of resveratrol supplementation. To summarize, face to the numerous human and animal studies that support the beneficial and protective properties of resveratrol, clinical studies that report on resveratrol’s harmful effects are few and controverted. Additionally, mechanisms underlying resveratrol’s molecular action need more investigation, walling for a more uniform design of clinical trials to properly investigate resveratrol’s therapeutic and preventive properties during pregnancy and related complications.

## 3. Resveratrol’s Mechanism of Action during Pregnancy Complications

**Figure 1:**
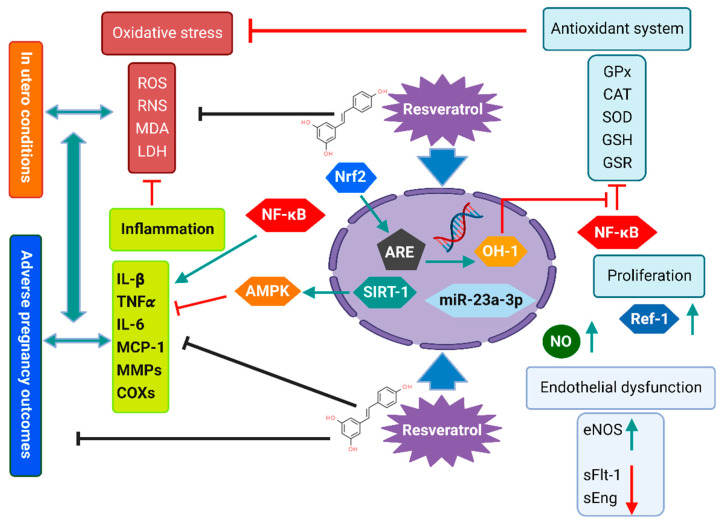
Potential molecular mechanisms underpinning the effects of resveratrol intake in mothers and their offspring in different models.

Interestingly, maternal resveratrol intake has been reported to have potential beneficial effects in adverse human pregnancies [3] and has been intensively studied in rodent models as a potential therapeutic agent in several pregnancy-related disorders, including pre-eclampsia (PE) [42], gestational diabetes mellitus (GDM) [22], fetal-growth restriction (FGR) [43], insulin resistance, and dyslipidemia [23,44]. However, the molecular mechanisms underlying its efficiency are not entirely understood [45]. Studies suggested that resveratrol’s potential protective mechanisms in adverse pregnancy outcomes are related to its pleiotropic properties, including antioxidant, anti-inflammatory, anti-obesogenic, anti-atherosclerotic, and anti-diabetic, which evidently associate its consumption with the prevention of several non-communicable diseases [20,46]. Figure 1 schematizes the potential molecular mechanisms that may underpin the protective effects of resveratrol intake in mothers and their offspring in different models.

A systematic data analysis across different species and dosages indicated that resveratrol consumption could decrease inflammation and oxidative stress in placental and embryonic tissues [3]. Oxidative stress is a phenomenon caused by increased levels of reactive oxygen species (ROS) resulting in disturbance of the cellular redox balance, which causes the impairment of cellular functions and eventually initiates pathological pathways leading to disease development [47,48]. It is now evident that oxidative stress is significantly higher during normal pregnancy compared to non-pregnancy periods in women [49]. In fact, the gestational and prenatal changes of the maternal body, as well as the high oxygen and energy required for fetal development, favor ROS overproduction [50]. These elevated ROS levels appear to be implicated in pregnancy-related disorders such as PE, intrauterine growth restriction (IUGR), and fetal death [51,52]. Thus, evaluating the redox status during pregnancy in order to find adequate strategies to counteract it appears to be an issue of utmost importance.

In this regard, studies in murine models have shown that oxidative stress biomarkers and metabolic dysfunction caused by a low-protein diet in the mother, placenta and offspring could be improved by a resveratrol-rich diet in pregnant individuals [53]. Anti-atherogenic activities, low fetal oxidative stress, and reduced apoptosis were also detected in rodent streptozotocin-induced diabetic models treated with resveratrol [54,55]. Moreover, increased risk of infertility is often linked to advanced maternal age, where the major oxidative stress-associated causative factors encompass decreased oocyte quality, low fertilization rate, poor embryonic development, low pregnancy rate, and a high rate of chromosomal aberrations, telomerase shortening, and apoptosis [56,57]. In this regard, by delaying cells’ aging process and preventing age-related diseases through ameliorating mitochondrial function and reducing ROS generation, several studies have reported that resveratrol supplementation can improve in vitro oocyte maturation and embryonic developmental competence in different species [58,59,60,61]. A study realized in a pregnant-aged mice model demonstrated that resveratrol intake increases blastocyst development, ameliorates pregnancy and implantation rates, and parallelly decreases ROS production with a significant increase in mitochondrial membrane potential, suggesting that resveratrol supplementation may improve pregnancy oxidative stress-mediated outcomes in women with advanced maternal age [62]. In this regard, Liu et al. [63] have also suggested that resveratrol can induce oocyte maturation and subsequent embryonic development in aged mice [63]. Indeed, aging-associated reproductive pathologies are frequently associated with impaired DNA repair, metabolic disorders, genomic instability, telomeric shortening, apoptosis, and mitochondrial dysfunction [58,64,65], which are the molecular target of resveratrol action.

Epigenetic regulation represents another potential mechanism related to the maternal resveratrol intake effects and modulates methylation and acetylation processes affect gene expression [66]. Among the main epigenetic changes exerted by resveratrol in the zygotic pronuclei are the methylation and acetylation of histone H3 on lysine 9 (H3K9). Gestational resveratrol supplementation was demonstrated to induce breast cancer-1 promoter (BRCA-1) hypermethylation and to decrease BRCA-1 expression in the mammary tissue of rat offspring [20]. Another mechanism through which resveratrol’s maternal intake may improve pregnancy outcomes is by relieving oxidative stress-related mitophagy [67,68]. Mitophagy is defined as the selective removal of dysfunctional mitochondria by autophagy, a process aberrantly affected by ROS increase and physiologically restored by oxidative stress balance [69]. Consonantly, a study by Zha et al. [70] demonstrated that resveratrol supplementation in pregnant mice might promote mammary gland proliferation and antioxidant activity through mitophagy activation [70]. Pregnancy-related metabolic disorders, such as GDM, occur when ROS prevents insulin from facilitating cellular glucose uptake, subsequently leading to insulin resistance [71]. Hypertensive disorders of pregnancy comprising chronic, white, mask and gestational hypertension, PE, hemolysis, elevated liver enzymes, and low platelet count were also found to be tightly related to altered pro-inflammatory and oxidative stress signals and be responsible for the increased risk of chronic diseases such as obesity, type 2 diabetes, and cardiovascular diseases in adult life [20,72,73,74].

Resveratrol is also recognized as a Sirtuin 1 (SIRT1) activator. The Sirtuin proteins family has a beneficial impact on longevity mainly linked to their effects on metabolic control since they are substantially contributing to lipid and glucose regulation via the deacetylation of crucial metabolic signals relevant to the activated.

The Sirtuin proteins family has a beneficial impact on longevity primarily related to their effects on metabolic control as they substantially contribute to lipid and glucose regulation through the deacetylation of crucial metabolic signals related to the activated protein kinase-SIRT1-PPARG coactivator-1α axis [75,76]. In this context, a study realized on rats exposed to a maternal high-fat diet during pregnancy, resveratrol supplementation (10 mg/kg) managed to restore the impaired expression of SIRT1, phosphor-extracellular regulated kinases1/2, (p-ERK1/2) and phospho-peroxisome proliferator-activated receptor γ (PPARγ), adiponectin and Brain-Derived Neurotrophic Factor (BDNF), all molecules involved in insulin resistance and mild cognitive dysfunction [77]. These previous data underline resveratrol’s ability to improve many maternal-associated complications including PE, FGR, diabetes, insulin resistance (IR), obesity, and metabolic syndrome [77].

## 4. Maternal Pregnancy-Related Disorders

### 4.1. Resveratrol Effect in Pre-Eclampsia and Related Disorders

Pregnancy is known to cause increased levels of oxidative stress, as an aspect of the systemic inflammatory response, resulting in high-circulated amounts of ROS, with the placenta representing the main source [78]. PE is a pregnancy-specific clinical syndrome characterized by a multifactorial physiopathology [79,80], that includes both new-onset of hypertension and new-onset proteinuria ≥300 mg/24 h after 20 weeks of gestation, most often near-term. However, when proteinuria registers normal levels, PE is considered thrombocytopenia-related hypertension, with altered liver function, renal insufficiency, pulmonary oedema, and new-onset cerebral or visual disturbances [81]. One condition that features oxidative stress during pregnancy is hypoxia, a common form of intrauterine stress defined as an abnormal exposure to low oxygen levels, resulting in an excessive generation of ROS [82]. Accordingly, increased placental hypoxia and uterine vascular resistance were revealed to be associated with PE and fetal/intrauterine growth restriction (FGR/IUGR) in several animal and human studies [83]. Gestational hypoxia induces ROS overproduction in uteroplacental cells’ mitochondria leading to oxidative stress [83]. In contrast, excessive ROS production causes uteroplacental dysfunction by altering cellular macromolecules, which underlies PE and FGR pathogenesis [83]. Resveratrol has been recently shown to exert a protective effect against prenatal hypoxia-induced programming of the metabolic syndrome when offspring-initiated treatment at weaning [84]. In a recent study, resveratrol intake (4 g/kg) managed to ameliorate adverse fetal outcomes in a rat model of severe prenatal hypoxia [28].

PE-associate oxidative stress originates from the placental impairment caused by hypoxia-reoxygenation imbalance, lower efficiency of both free radical scavengers and antioxidant enzymes, impaired angiogenesis, decreased nitric oxide (NO) bioavailability vascular endothelial dysfunction, cardiovascular complications, and exaggerated inflammatory response [85,86,87,88,89]. A poorly perfused fetoplacental unit releases free radicals and initiates oxidative stress impairment in placental cells [88,90,91]. Resveratrol was demonstrated to activate endothelial nitric oxide synthase (eNOS) and increase NO and nuclear factor-erythroid-derived 2-related factor-2 (Nrf2) production [92]. In response to resveratrol, Nrf2 binds to the antioxidant response element (ARE) promoter upregulating the expression of antioxidant proteins, including heme oxygenase-1 (HO-1) and glutathione reductase (GSR), thus counteracting oxidative stress and balancing the cellular redox state [93,94]. Indeed, in an in vitro PE model, resveratrol increased ARE activity and reduced ROS in endothelial cells exposed to plasma from PE patients compared to cells exposed to plasma from healthy pregnancies. Noteworthy, plasma from PE patients obtained 1 hr after the ingestion of polyphenol-rich whole red grapefruit juice (rich in resveratrol) significantly increased NO production and reduced antioxidant markers in the exposed cells compared with the serum before juice intake [94].

PE etiology is not fully understood, however, this condition is believed to be associated with impaired uterine artery blood flow [95]. In this context, recent studies have shown that resveratrol can induce uterine arteries relaxation during PE pathological events. Interestingly, resveratrol supplementation with a dose of 4 g/kg was demonstrated to ameliorate the PE-like phenotype by significantly increasing uterine artery blood flow velocity and fetal weight in pregnant C57BL/6J, eNOS⁻/⁻, and COMT⁻/⁻ mice murine models, suggesting resveratrol consumption as a potential therapeutic strategy for PE disorder [95].

It has been recently shown that circulating SIRT1 is reduced during PE [96]. In this regard, a group of researchers investigated the effects of resveratrol as a SIRT1 activator using the previously reported in vitro/clinical PE model compared to gestational hypertensive (GH) and healthy pregnant (HP) women. The authors showed that resveratrol-elicited SIRT1 activation alone did not exert any effect and may not be beneficial to women with PE, suggesting that pregnant women with PE may have different responsive mechanisms to this molecule [96]. Furthermore, aberrant trophoblast invasion is among the factors involved in PE occurrence and development since it contributes to the progression of multiple conditions and is characterized by impaired spiral artery remodeling [97], cell-matrix restructuring, and cytoskeleton dynamics [98], during which epithelial-mesenchymal transition (EMT) occurs leading to the breakdown of cell–cell adhesion, the loss of epithelial phenotypes, and cell depolarization [99,100]. During pregnancy, the acquisition of invasive phenotypes by the extra-villous trophoblast (EVT), was found to be involved in PE pathogenesis and was suggested to be linked to EMT [101]. In this regard, using both in vitro (HTR-8/SVeno cell culture) and in vivo (NG-nitro-l-arginine methyl ester mice model) PE models, Zou et al. [102] suggested that resveratrol (100 µmol/L resveratrol /20 mg/kg/day) might stimulate the invasive capability of human trophoblasts by promoting a Wnt/β-catenin pathway-mediated EMT transformation, increasing trophoblasts migration and the invasion in the cell model, and markedly ameliorating hypertension and proteinuria in mice [102]. Molecularly, resveratrol-mediated EMT activation occurred through the regulation of E-cadherin, β-catenin, N-cadherin, and vimentin expression, and altered the WNT-related gene expression, including WNT1, WNT3, and WNT5B [102].

Several studies suggested that the resveratrol-associated anti-hypertensive effect is mainly related to its capacity to attenuate blood pressure and hypertension symptoms. Interestingly, a clinical trial with 400 PE patients investigated the outcome of a nifedipine/resveratrol combined treatment against PE, revealing that resveratrol supplementation significantly reduced the time needed to control blood pressure with a better result in the group treated with both nifedipine (10 mg, up to 5 dosages) and resveratrol, which also had a significantly delayed time between the hypertensive crises intercourse (50 mg, up to 5 dosages) [23]. In contrast, resveratrol did not produce any apparent effect on rats in the study by Moraloglu et al. [42], where a Desoxycorticosterone acetate (DOCA) hypertension PE model was used to test the potential hypotensive effect of resveratrol on the blood flow. Interestingly, DOCA managed to increase blood pressure and placental and renal blood flow levels, whereas, no significant differences for the same parameters were rated in groups treated with resveratrol (20 mg/kg per day) [42].

Consistent with this, other studies indicated that resveratrol anti-hypertensive properties are also related to its ability to inhibit the release of endothelial dysfunction-associated anti-angiogenic factors and that their elevation is a main player in placental oxidative stress and inflammation during the maternal endothelial dysfunction in PE [103]. For instance, serum soluble fms-like tyrosine kinase-1 (sFlt-1), also known as a soluble receptor for vascular endothelial factors (VEGF), is a protein that binds and decreases the concentrations of circulating VEGF and placental growth factor (PlGF) [104,105]. While Endoglin (Eng) is a transmembrane glycoprotein and is considered another factor playing a role in PE etiology and an accessory receptor for the transforming growth factor-beta (TGF-β). Eng is highly expressed in syncytiotrophoblast and proliferating endothelial cells [106], it influences the TGF-β and eNOS signaling pathways resulting in a significant angiogenesis modulation [107]. In this regard, a study carried out in vitro by Hannan et al. [108] demonstrated that resveratrol (0–100 μM) decreases sFlt-1 and soluble Eng secretion from primary trophoblasts and human umbilical vein endothelial cells (HUVECs), a phenomenon that positively correlated with increased mRNA expression of the pro-inflammatory molecules NFκB, IL-6, and IL-1β in the trophoblast and decreased of IL-6, IL-1β, and TNF-α [108]. Additionally, resveratrol significantly increased the mRNA expression of several antioxidant enzymes including HO-1, NADPH Quinone Dehydrogenase (1NQO1), Glutamate-Cysteine Ligase Catalytic (GCLC) subunit and thioredoxin (TXN) in HUVECs, while reducing HO-1 protein levels in trophoblast cells. Expression of the cell adhesion molecule VCAM-1 and the adhesion of peripheral blood mononuclear cells were also increased by resveratrol supplementation [108]. Further, TNF-α-induced endothelial dysfunction in HUVECs was significantly ameliorated by resveratrol through i) the reduction in TNF-α-induced Endothelin-1 (a vasoconstrictor) expression and ii) the increase in endothelial eNOS phosphorylation [108]. Resveratrol (20 mg/kg/day) has also been reported to prevent cells from p53- and ROS-dependent apoptosis induced by IL-1β in an NG-Nitro-l-arginine methyl ester-induced PE rats model via the increase in superoxide dismutase (SOD) suggesting that resveratrol significantly opposes oxidative stress effects in vivo [109]. Overall, resveratrol can be suggested to alleviate PE symptoms and decrease its effects in pregnant individuals and their offspring in different models via multiple pathways mainly related to the attenuation of vascular injury, placental dysfunction, hypertension symptoms, anti-angiogenic factors, oxidative stress and inflammatory responses Table 1 Taken together, the reported data suggests resveratrol as a potential therapeutic tool in PE.

### 4.2. Resveratrol Effect in Gestational Diabetes and Related Metabolic Disorders

GDM is a type of diabetes characterized by an impaired glucose tolerance that occurs during pregnancy [110]. Although GDM is a reversible pathology and the impaired metabolism returns to normal after delivery, the risk of developing type 2 diabetes later is high [111]. GDM is extremely harmful to mothers and their offspring and may induce PE, premature rupture of membranes, and premature delivery [112]. The main features of GDM during pregnancy include increased glucose demand, increased insulin resistance, and relative insufficiency in insulin secretion [113]. Recent studies emphasized the protective effects of resveratrol in diabetes and its related-cardiovascular complications, involving the regulation of multiple signaling pathways, inhibition of oxidative stress and inflammation, enhancement of insulin sensitivity, induction of autophagy, regulation of lipid metabolism, promotion of GLUT4 expression, translocation, and activation of SIRT1/AMPK signaling axis [114]. Several studies have demonstrated that resveratrol lowers maternal blood sugar levels, ameliorates the maternal lipid profile [115], and prevents delays in embryo development in rat diabetic dams models [54]. In a recent study performed in streptozotocin GDM pregnant rat model, the ability of resveratrol to lower blood glucose and blood lipids was tested. Resveratrol supplementation demonstrated a dose-dependent effect (120 and 240 mg/kg), which positively correlates with a decrease in both insulin and blood glucose levels in comparison with the control group; remarkedly demonstrating a stronger effect than metformin hydrochloride in improving GDM outcomes [116]. Using the same previous model, it was reported that the resveratrol-zinc oxide complex encapsulated with chitosan (CS-ZnO-RS) (an encapsulated form of resveratrol to increase the stability and effectiveness of substances in gestational diabetes management), significantly decreased the blood glucose levels and maintained the lipid content similar to control group levels, while simultaneously reducing the level of pro-inflammatory factors (IL-6 and MCP-1) and significantly decreasing endoplasmic reticulum stress components (GRP78, p-IRE1α, p-eIF2α, and p-PERK), and further inhibiting α-glucosidase and α-amylase activity in a dose-dependent fashion [117].

Studies also demonstrated that GDM is often related to adverse metabolic health outcomes in offspring as a common pregnancy complication. In a GDM model of female Sprague Dawley rats fed with a high-fat and sucrose diet, Brawerman et al. [118] showed that maternal resveratrol supplementation (147 mg/kg/day) protects against GDM-induced glucose intolerance and offspring islet dysfunction by restoring glucose tolerance and normoglycemia and improving insulin secretion. At 15 weeks of age, hepatic steatosis, IR, glucose intolerance, and dysregulated gluconeogenesis were attenuated in offspring of resveratrol-treated dams [118]. Moreover, the dysregulation of several metabolic genes (e.g., lpl: lipoprotein lipase; ppara: peroxisome proliferator-activated receptor α; g6p: glucose-6-phosphatase) were also attenuated whilst glucose-stimulated insulin secretion was improved in the offspring islets of resveratrol-treated dams [118].

It is well known that insulin resistance (IR) caused by insufficient insulin secretion during pregnancy, may lead to metabolic disorders, mainly GDM and related pathologies [119]. Attention was recently given to the role of resveratrol in ameliorating IR in pregnancy-related metabolic disorders. Consistently, recent studies have shown that resveratrol may improve glucose uptake in adipocytes with IR, ameliorate IR in mice fed with a high-fat diet, and enhances glucose metabolism and insulin tolerance in GDM mice (100 mg/kg) [120,121]. MicroRNAs (miRNAs), a type of short-chain non-coding endogenous RNAs existing in eukaryotic organisms, are known to exert regulatory effects on several cell functions and play a different role in many diseases [122,123,124,125,126]. Recent studies confirmed the resveratrol’s capacity to regulate their expression [16], especially that of MiR-23a-3p, which was reported to be low-expressed in adipose tissues of obese and diabetic patients; in fact, its over-expression can reduce TNF-α-induced IR in adipocytes [127]. In a study realized on a high-fat diet GDM mice model, resveratrol supplementation (0.2%) managed to ameliorate glucose uptake and lipid metabolism, regulating the miR-23a-3p/NOV axis through the increase in Adiponectin, Leptin, Phosphoinositide 3-kinase (p-PI3K), and phosphorylated Akt (p-Akt) in adipocytes with IR and parallelly decreasing the nephroblastoma (NOV) overexpression [128]. In diabetic embryopathy-murine models, resveratrol (50 mg, up to 5 dosages) significantly ameliorates the embryonic outcome in terms of diminishing developmental abnormalities, a phenomenon likely associated with its antioxidative potential, anti-diabetic action, and anti-dyslipidemic nature [23].

Maternal obesity is also a metabolic complication that rates high maternal and fetal oxidative stress and inflammation. Hypothetically, offspring of obese mothers are at greater risk of developing obesity, diabetes, and cardiovascular disorders in adult life [129], with risk factors that include variations in both maternal glucose and lipid metabolism (predisposition to GDM in particular), abnormal pregnancy hormone concentrations [130,131,132], stillbirth, premature birth, and macrosomia [132,133].

A recent study conducted in obesity murine models revealed that maternal resveratrol supplementation (20 mg/kg/day) improves maternal metabolism and reduces the placental and liver oxidative stress of mothers and fetuses in a sex-dependent manner [134]. Compared to the untreated group, resveratrol supplementation in pregnant animals was able to lower adipocytes number, triglycerides serum concentrations, insulin resistance, liver fat accumulation, expression of genes related to insulin resistance, inflammatory processes, and lowered oxidative stress in mothers, placentas, and female fetal liver [134]. Resveratrol treatment (5 mg/kg/day) was shown to improve some of the altered metabolic symptoms in a programmed prenatal and postnatal high-fat exposure in the progeny of Sprague Dawley dams’ experimental model, including peripheral leptin resistance, and related dysbiosis of the gut [135,136]. Another study suggested that resveratrol’s protective effects during pregnancy and lactation are diet dependent, where resveratrol supplementation (2.0 to 2.5 mg/kg/d/dam) decreased body weight and adipose tissue content in offspring of dams on a high-fat diet but did not affect offspring from the low-fat diet-fed dams [137]. In addition, resveratrol supplementation (300 mg/kg) was demonstrated to increase high-density lipoprotein cholesterol and low-density lipoprotein cholesterol in the plasma and partially improved the fat metabolism in the adipose tissue in piglets’ experimental model [138]. Intrahepatic cholestasis of pregnancy (ICP), is a pregnancy-specific liver disease characterized by raised serum bile acids and adverse fetal outcomes partially related to SIRT1 dysregulation [139]. Liao et al. [139] studied the molecular and biochemical mechanism underpinning resveratrol’s regulation of the SIRT1- nuclear factor-κB (SIRT1-NF-κB) signaling pathway and bile acid biosynthesis in ICP. Resveratrol (30–120 mg/kg/day) was demonstrated to decrease bile acid levels in the ICP rat model and was suggested to protect syncytiotrophoblast against trichloroanisole (TCA)-induced inflammatory injury through the upregulation of SIRT1 and the downregulation of RelA/p65, a subunit of NF-κB recognized as an activator of SIRT1 [139]. In a related study, it was shown that resveratrol ameliorates ICP conditions by downregulating the overexpression of matrix metalloproteinases (MMPs) [140]. Indeed, in the ethinylestradiol-induced ICP rat model, resveratrol-diet (15 mg/kg/day) was able to inhibit the elevation of both MMP-2 and MMP-9, and exhibited better outcomes in restoring bile flow rate, serum enzymatic activities, and total bile acids (TBA) concentration compared to the ICP known drug ursodeoxycholic acid [140].

On the other hand, diabetes-associated lipid profile disruption may indirectly affect embryogenesis and organogenesis [141], and the impairment of the couple glucose levels/lipid status may simultaneously alter the fetal and neonatal growth; which may later impact offspring life and contribute to adult obesity. Using the same previous GDM model, Singh et al. [22] evaluated the effect of resveratrol on lipidic profile variations. Compared with the untreated GDM group, resveratrol supplementation demonstrated a dose-dependent effect (120 and 240 mg/kg), which significantly increased both HDL and adiponectin levels and inversely correlated with the levels of leptin, resistin, TNF-α, IL-6 levels, the body weight, total cholesterol (TC), triglycerides (TG), and low-density lipoprotein (LDL) [22]. Parallelly, resveratrol was shown to reduce mRNA levels of 3-hydroxy-3-methylglutaryl-CoA reductase (HMG-CoA reductase), the rate-limiting enzyme of cholesterol synthesis and the statin drugs target in high-fat fed hamster model [142]. Statin is an active drug that is used to lower human cholesterol and triglycerides but may be harmful to pregnant or nursing women [143]; therefore, in light of these findings, the authors suggested resveratrol as a potential therapy for an altered lipidic profile during pregnancy-associated metabolic disorders.

Overall, the above-mentioned studies evaluated the short-term effects of maternal resveratrol intake in diabetic and related-metabolic disorders experimental models Table 2. Resveratrol showed favorable results on metabolic homeostasis and redox state. Nevertheless, the mother and offspring’s longer-term outcomes remain poorly specified. Not to mention the variations of experimental models, range of doses, routes of administration and therapeutic periods that may contribute to inconclusive results regarding the potential metabolic benefits of maternal resveratrol supplementation during pregnancy and lactation. In this light, extended studies with well-established experimental models are needed.

## 5. Fetus-Related Abnormalities and Resveratrol

### 5.1. Prenatal and Resveratrol

Several studies supported resveratrol’s protective and therapeutic effects at the prenatal stage. In fact, offspring may show increased susceptibility to certain diseases due to prenatal or intrauterine conditions, which may be efficiently reversed by resveratrol. In this section, we present several examples of disease susceptibility due to intrauterine modifications and the potential role of resveratrol in reversing this susceptibility. These include susceptibility to metabolic, cardiovascular, and neurological disorders.

Non-alcoholic fatty liver disease (NAFLD) is a hepatic metabolic disorder [145] associated with IUGR [146]. A study showed that prenatal ethanol exposure (PEE) increases the susceptibility to NAFLD in female IUGR rat offspring by inducing intrauterine metabolic alterations [147]. These alterations enhance fetal hepatic lipogenesis and reduce lipid output in utero; moreover, the fetuses of PEE pregnant rats showed decreased weight and low levels of serum glucose and triglycerides, in addition to hepatocellular ultra-structure modifications [147]. Further analyses also showed attenuated SIRT1 expression and activity associated with the upregulation of SREBP1c and other downstream lipogenic genes, such as fatty acid synthase (FASN), acetyl-coenzyme A carboxylase α (ACCα), and stearyl-coenzyme A desaturase 1 (SCD1) [147]. Interestingly, the hepatic SIRT1-SREBP1c signaling pathway has been reported to play a crucial role in controlling hepatic lipid metabolism and thus promoting liver protection against NAFLD [148]. Accordingly, by targeting the hepatic SIRT1-SREBP1c pathway, the SIRT1 chemical activator resveratrol (50 mg/kg/day) was able to reduce the increased susceptibility to diet-induced NAFLD by reversing the intrauterine programming of hepatic lipogenesis after birth in PEE offspring rats [149]. Another study showed that a maternal high-fat (HF) diet affects the offspring of Sprague Dawley and may lead to metabolic dysregulation [136]. Results showed that offspring dams with maternal HF exposure have decreased acetate propionate and butyrate levels in plasma. In addition, the gut microbiota metagenome of these offspring was altered, thus altering their metabolic homeostasis. The study also suggested that this metabolic dysregulation in offspring was programmed intrauterine by the maternal HF diets and is related to their gut microbiota [136]. In the same study, resveratrol treatment (10 mg/kg/day) was able to reverse the effects induced by maternal HF diet exposure and ameliorate plasma propionate levels. In addition, resveratrol relieved metabolic syndrome dysregulation and related dysbiosis of gut microbiota [136].

Offspring susceptibility to hypertension may originate at the prenatal stage due to maternal conditions. A study showed that the HF diet exacerbated maternal L-NAME treatment-induced programmed hypertension of rat offspring [150]. This was associated with increased oxidative stress, attenuated AMP-activated protein kinase (AMPK)/ peroxisome proliferator-activated receptor γ co-activator 1α (PGC-1α) pathway and altered gut microbiota [150]. These alterations were initiated at the prenatal stage by induced hypertensive maternal condition, sustained till adulthood, and exacerbated by the HF diet. Resveratrol (50 mg/L in drinking water) reversed the increase in Firmicutes to Bacteroidetes ratio in the gut microbiota species while amplifying the abundance of phylum Verrucomicrobia and genus Akkermansia [150]. The overall data indicate resveratrol may attenuate hypertension of developmental origin by regulating the microbiota.

It is accepted that prenatal hypoxia increases offspring susceptibility to cardiovascular disease and metabolic disorders [151,152] such as altered cardiac morphology and dysfunction and metabolic complications in adulthood [153,154,155]. Furthermore, prenatal exposure to hypoxia was reported to set the stage for ischemia/reperfusion (I/R) injury [156,157] and metabolic syndrome in response to a secondary insult such as the HF diet [84]. Interestingly, prenatal hypoxia is able to compromise metabolic and cardiac function in a sex-specific manner [158]. Accordingly, prenatal hypoxia and the HF diet impaired metabolic function in the male but not in female offspring rat [158]. In addition, prenatal hypoxia compromised I/R injury recovery more profoundly in the male compared to female HF diet rat offspring [158]. These prenatally-programmed effects were alleviated by resveratrol administration. Indeed, resveratrol (4 g/kg diet) improved cardiac recovery and attenuated oxidative stress in both male and female rat offspring, thus, impeding their increased susceptibility to cardiovascular diseases [84]. In addition, resveratrol (D120020402 4 g kg^−1^; Research Diet) improved their metabolic function prenatally challenged by hypoxia and postnatally by HF diet [158]. Molecularly, resveratrol enhanced cardiovascular and metabolic function by mitigating oxidative stress and activating the AMP-activated protein kinase (AMPK)–acetyl CoA carboxylase (ACC) and AMPK/perioxysome proliferator-activated receptor-γ coactivator (PGC)-1α pathways, resulting in improved fatty acid oxidation, mitochondrial biogenesis, and metabolic and cardiac health [158].

Prenatal stress may predispose neurological complications in offspring. In a study realized on rat offspring, prenatal gestational stress increased lipid peroxidation and protein oxidation and decreased antioxidant activity and NO levels [159]. In addition, their dentate gyrus and CA3 hippocampal neurons were severely affected [159]. Resveratrol administration (10 mg/kg) alleviated prenatal stress-induced oxidative stress and the recovering neurons of the dentate gyrus [159]. Thus, resveratrol antioxidant potential is suggested to dispose of a neuroprotective role against prenatal stress-induced oxidative damage in neonatal rat brains [159]. In another study conducted by Sahu et al. [160], resveratrol prenatal-intake effect against cognitive deficit in rat offspring was investigated. Prenatal stress exposure managed to deteriorated spatial learning and memory and reduced Na(+), K(+)-ATPase activity in the rats’ offspring brains, while resveratrol administration (10 mg/kg) did not affect ATPase levels and it improved their spatial learning and memory, suggesting a neuroprotective efficacy of resveratrol against prenatal stress-induced cognitive impairment [160].

Autism spectrum disorder (ASD) is a neurological disorder characterized by decreased social communication and interaction [161]. While little is known about ASD causes, recent evidence supported its association with estrogen receptor β (ERβ) dysregulation [162,163]. In fact, maternal hormonal exposure to ERβ inhibitors such as natural progesterone and synthetic progestin impairs cognitive flexibility and contributes to ASD development [164,165]. A study showed that prenatal exposure of rat offspring to progestins leads to decreased ERβ expression in the amygdala [166]. This downregulation was concomitant with an autism-like behavior in the offspring. These prenatal progestin-induced biochemical and behavioral alterations were shown to be reversed by resveratrol administration (20 mg/kg) [166]. Resveratrol supplementation also reduced progestin-induced oxidative stress, mitochondrial dysfunction, and lipid dysregulation in the brain, through the activation of ERβ and its target genes leading to the amelioration of progestin-induced ASD-like behavior effects [166]. These results are promising for the protective effect of resveratrol against progestin intake, be it clinical such as oral contraceptives, or dietary such as in contaminated water and seafood, suggesting that resveratrol may limit the risk of ASD; however, more clinical studies are needed to determine the resveratrol doses and duration of treatment in pregnant women. Collectively, these data support the protective role of resveratrol against a prenatally-induced predisposition to cardiovascular, neuronal, and metabolic pathological conditions. This protective effect appears to be mainly associated with resveratrol antioxidative properties.

### 5.2. Placenta and Resveratrol

The placenta is a large organ that develops in the uterus during pregnancy. It provides oxygen and nutrients to the fetus and removes waste products from its blood. It plays a crucial role in fetal organ development and fetal growth [167]. In addition, the umbilical cord of the fetus arises from the placenta [168]. Assessing placenta oxidative status as oxidative stress indication during pregnancy is important due to two main reasons. First, the placenta is the interface between the mother and fetus [167]. Changes in oxygen levels in the placenta to support increased metabolic rate are associated with increased circulating ROS [169]. This redox imbalance in the placenta may have a direct effect on fetal development. The second reason is that the placenta exacerbates pregnancy inflammation by producing inflammatory factors [170,171,172,173]. Thus, it is an important site for the propagation of maternal inflammation.

Noteworthy resveratrol administration during pregnancy ameliorates placental oxidative status, which is achieved through a plethora of biological effects [167,174,175,176]. These resveratrol-initiated effects have been particularly shown to alleviate chemical, microbial, heavy metal, and high-fat diet (HFD)-induced inflammation in the placenta. Consistently with that, resveratrol suppressed cadmium (Cd)-induced expression of inflammatory cytokines and chemokines in the placenta of pregnant CD-1 mice and JEG-3 cells (0.2%) [177]. These include tumor necrosis factor-α (TNF-α), interferon-gamma (IFN-γ), monocyte chemoattractant protein 1 (MCP-1), macrophage inflammatory protein-2 (MIP-2), and chemokine (C–X–C motif) ligand 1 (KC) [177]. In contrast, a study conducted on unchallenged pregnant sows failed to report any influences of dietary resveratrol (300 mg/kg) on pro-inflammatory cytokine levels such as interleukin 1β (IL-1β), IL-6, IL-8 and tumor necrosis factor α (TNF-α) [167]. This discrepancy may be due to the dose variation and duration of resveratrol and stimulus administration (Cd versus no stimulus). It is also worth mentioning that in the latter study, resveratrol played an antioxidative rather than an anti-inflammatory role. In another study realized in the human placenta, resveratrol treatment (200 µM) significantly attenuated IL-1α, IL-1β, IL-6, and IL-8 mRNA expression and reduced the release of IL-6, IL-8, and MCP-1 [173]. Interestingly, these anti-inflammatory effects were observed when the human placenta was stimulated with TNF-α, IL-1β, or the synthetic viral dsRNA analogue polyinosinic: polycytidylic acid (poly (I:C)) [173]. Effectively, several studies support the resveratrol antioxidant effect at a placental level. For example, a study showed that resveratrol treatment (0.2 %) attenuated Cd-induced upregulation of endoplasmic reticulum (ER) stress markers in the placenta of pregnant mice [177]. A similar antioxidant effect of resveratrol (300 mg/kg) was reported in the placenta of pregnant sows [167] where resveratrol was able to increase the expression of Nrf2 and decreased the expression of Kelch-like ECH-associated protein-1 (Keap1) [167]. In addition, resveratrol increased the mRNA expression of antioxidant enzyme genes including catalase (CAT), glutathione peroxidase 1 (GPX1), SOD1 and HO-1. Furthermore, phase 2 detoxification genes, including glutamate-cysteine ligase modifier (GCLM), microsomal glutathione S-transferase 1(MGST1) and UDP glucuronosyltransferase family 1 member A1 (UGT1A1) were upregulated by resveratrol administration (300 mg/kg) [167].

The placenta favors the flow toward the fetus of epigenetic alterations induced by the mother’s conditions, such as GDM and obesity [178,179]. These epigenetic alterations may induce pregnancy complications, affect fetal and neonatal phenotype and induce offspring disease susceptibility [178,179]. In this regard, resveratrol elicits an anti-epigenetic activity by regulating epigenetic enzymes. For instance, resveratrol attenuated both DNA methyltransferase (DNMT) activity and DNMT3B expression in the placenta of Cd-challenged pregnant mice (0.2 %) [177]. In addition, resveratrol administration enhanced the activity and expression of the nuclear deacetylase, Sirtuin 1 (SIRT1), and inhibited Cd-induced PI3K/Akt signaling pathway [177]. A similar activating effect was elicited by resveratrol on the Sirt1 protein in the placenta of pregnant sows (300mg/kg) [167]. Furthermore, resveratrol (100 µmoL/L) has been shown able to prevent the up-regulation of the placental early growth response protein-1 (Egr-1), which is a protein that regulates DNA demethylation [180]. Although maternal nutritional epigenetics is an intriguing topic under intense investigation [181], further studies are necessary to better understand the role of resveratrol in modulating maternal epigenetics.

Studies on non-human primates have also shown that resveratrol reduced placental inflammation and improved glucose metabolism [39,144]. A study by Tran et al. [173] showed that resveratrol (200 µM) restored insulin signaling and glucose uptake aberrantly altered by the pro-inflammatory cytokines TNF-α and IL-1β, the bacterial lipopolysaccharides LPS and polyinosinic-polycytidylic acid (poly(I:C) in human placenta tissue [173]. Not only did resveratrol alleviate placental inflammation, but it also reduced insulin resistance in the human placenta tissue [173]. As previously stated, the release of endothelial dysfunction-associated anti-angiogenic factors such as sFlt-1 is one of the main features in placental oxidative stress and inflammation during PE [103,180]. In humans, resveratrol (100 µmoL/L) abolished cytokine-induced release of sFlt-1 from normal and from pre-eclamptic placental explants [180]. A similar inhibitory effect on sFlt-1 was observed in resveratrol-treated HUVEC or HTR-8/SVneo [180]. In these cells, resveratrol also increased the expression of HO-1, an enzyme found to be dysfunctional in preeclampsia [180]. These latest studies indicate that resveratrol plays anti-inflammatory, antioxidative, and growth factor-regulating roles at the level of the placenta by modulating oxidative/antioxidative enzymes expression, counteracting proinflammatory cytokines release and modulating the expression of growth factors concentration such as VEGF and PlGF through sFlt-1 [104,105].

Overall, the presented data indicate that dietary resveratrol supplementation during pregnancy improves the placental status, which is beneficial for reproductive performance and the offspring’s well-being. However, long-term clinical trials on pregnant women are warranted to reinforce resveratrol’s beneficial effects on the placenta and placenta-associated functions.

### 5.3. Preterm and Resveratrol

Preterm birth (PTB) (<37 weeks of gestation) is considered a leading cause of newborn death and a risk factor for short and long-term adverse health outcomes. Although the etiology of PTB remains unclear, it is thought that an inappropriate increase in net inflammatory load seems to be a pivotal key [182]. Genital tract infections by Gram-negative bacteria are a common complication in human pregnancy and have been shown to increase the risk of preterm delivery. Several studies demonstrated that bacterial Lipopolysaccharide (LPS) elicits a strong maternal inflammatory response that often results in preterm delivery and fetal death in the murine model endotoxin-induced preterm labor [183]. Such a phenomenon has been reported to occur via the induction of cytokine/chemokine production and infiltration of uterine, placental, and fetal tissues with leukocytes, leading to the release of prostaglandins, endocannabinoids, nitrogen, ROS, and MMPs [184]. Interestingly, prostaglandins, NO and TNF-α components were demonstrated to trigger preterm labor in both animal models [185,186,187] and humans [188]. The endocannabinoid system (ECS) is a cell-signaling system found in multiple organ systems and is integral to sustaining the microenvironment necessary for early pregnancy success and maintenance. A great body of literature suggests that in addition to early pregnancy events, the ECS plays a major role in regulating pregnancy maintenance and labor timing [189]. Through the ECS and PPAR-α systems modulation, resveratrol pre-treatment was found to prevent oxidative stress and inflammation by increasing the tissue levels of palmitoylethanolamide (PEA) and cannabinoid receptors type 1 CB1 and type 2 CB2 in sham animals. In the same model, resveratrol also induces a general increase in PPAR-α and COX-2 protein levels, thus attenuating inflammation and oxidative stress and leading to PTB prevention [190].

Despite the immensity of research into PTB pathophysiology, the resveratrol administration effect on this disorder remains unclear, although few studies suggested that resveratrol decreases PTB rate mainly via the suppression of inflammation and infection complications. Regarding the studies included in this review, only a few investigated the correlation between resveratrol consumption and PTB. In a study realized by Bariani et al. [183], in vivo treatment of 15-day pregnant BALB/c mice with resveratrol (3 mg/kg) prevented the LPS-induced PTB in 64% of the cases, whereas only 15% of mice in the LPS treated group had normal term birth. Resveratrol’s treatment resulted in a reduced NOS activity (*p* < 0.05) in the uterus of LPS-treated mice and parallelly reduced the expression of LPS-induced pro-inflammatory agents, such as iNOS (*p* < 0.05), COX-2 (*p* < 0.05), prostaglandin E2 (PGE2) (*p* < 0.05), and anandamide (AEA) (*p* < 0.05). Interestingly, resveratrol administration also prompted ameliorative changes in the LPS-altered uterine endocannabinoid profile [183]. In addition, spontaneous PTB is tightly linked with underlying intrauterine inflammation and infection that evokes an immune response involving the release of cytokines such as IL-1β, TNF-α, [191,192,193,194], cyclooxygenase (COX)-induced PGs [195], and matrix-degrading enzymes [196], which trigger uterine contractions, membrane rupture, and cervical ripening [197], leading to the infant preterm delivery. In Furuya et al.’s research [198], resveratrol administration (20 to 40 mg/kg) significantly decreased the PTB rate in an LPS-induced preterm mouse model and LPS-exposed peritoneal macrophages by suppressing the increased proinflammatory cytokines and consequent elevation of macrophages COX-2 [198]. Moreover, resveratrol uptake abolished TNF-α and interleukin IL-1β activity by downregulating their expression without any apparent effect on IL-6 levels. Parallelly, resveratrol treatment suppressed the elevation of TNF-α and IL-1β levels in LPS-exposed peritoneal macrophages and significantly eradicated the proinflammatory cytokine-mediated elevation of COX-2 in peritoneal macrophages [198].

It is well established that SIRT1 is mainly localized in the nucleus to exert its antiapoptotic effects during oxidative stress or toxic substance-induced damage, and its amount increases in the nucleus to protect the cell from apoptosis. In a tentative to identify the protective role of SIRT1 in PTB and the role of resveratrol to ameliorate its dysregulation, a study realized on PBMCs cells sampled from an established hyperoxia model of premature infants administered with different amounts of oxygen at birth, demonstrated a concentration-dependent increase in SIRT1 translocation rates, ROS and MDA levels. Interestingly, resveratrol (6 µmol/L) treatment managed to attenuate hyperoxia-associated outcomes resulting in opposite effects [199].

Overall, the effects of resveratrol in PTB complications appear to be based on its capacity to counteract infections, inflammation, and oxidative stress-related outcomes. Nevertheless, further investigations including clinical trials are needed to better elucidate the molecular underlying mechanisms of action related to the beneficial effect of resveratrol intake on PTB.

### 5.4. Fetus and Resveratrol

The “fetal basis of adult disease” hypothesis is now considered a keystone in the development of important diseases in adult/advanced age, such as cardiovascular and metabolic diseases. These events are associated with epigenetic dysregulations, and they are mainly supported by alterations in the fetus development during pregnancy and caused by non-optimal conditions in the uterus, such as GDM, maternal malnutrition, and functional placental deficit [200,201].

Resveratrol pregnancy intake appears to be able to reverse the negative effects on the fetus caused by in-utero adverse conditions and induce a positive impact on adult life health. Although studies have been carried out on different species and with different dosages and routes of administration, the overall data indicate that resveratrol is capable of providing positive effects on fetal development in different gestational phases [3].

Prenatal exposure to environmental stress can also lead to greater susceptibility to clinical disorders in adulthood. This is the case of fetal exposure in utero to 2,3,7,8-tetrachlorodibenzo-p-dioxin (TCDD) leading to alterations in the differentiation of T cells in the thymus and to a great susceptibility to autoimmune diseases [202]. TCDD can trigger toxicity through activation of the aryl hydrocarbon receptor and severely affects maternal and fetal immune systems during pregnancy [203]. A study performed in mice by Singh et al. [204] evaluated whether resveratrol administration (100 mg/kg of body weight) inhibited TCDD-induced immunotoxicity during pregnancy in the mother and fetus. Resveratrol was observed to protect not only normal non-pregnant mice, but also pregnant mothers and their fetuses from TCDD-induced thymic atrophy, apoptosis, alterations in T cell receptor, molecule expression, and T cells differentiation stimulatory. Furthermore, Cytochrome P450 Family 1 Subfamily A Member 1 (CYP1A1) expression in the thymus of both mother and fetus is significantly reduced when resveratrol is used in vivo after exposure to TCDD. Taken together the results demonstrate that resveratrol consumption, during pregnancy, is able to protect the mother and the fetus from toxicity induced by environmental pollutants such as TCDD [204].

Mothers exposure to ethanol during pregnancy may also induce various disorders in their offspring, known as fetal alcohol spectrum disorders (FASD), which mainly affect the cerebellum from developing. A study by Kumar et al. [205] demonstrated that resveratrol exhibited neuroprotective and antioxidant effects in the cerebellum of a rat pups model exposed to ethanol by acting on redox regulatory proteins. In particular, resveratrol administration managed to inhibit apoptosis and increase cerebellar granule cell survival. On a molecular level, resveratrol was able to restore ethanol-induced Nrf2 variation levels and parallelly maintained physiological levels of the expression and the activity of its downstream gene targets, such as NADPH quinone oxidoreductase 1 and superoxide dismutase in the same experimental model [205].

Several benefits of resveratrol administration (1 g total trans-resveratrol was implanted subcutaneously into the animal) during pregnancy and fetal development were notable in the study by Darby et al. [206], where they showed its crucial role in increasing uterine artery blood flow in pregnant sheep, leading to increased fetal growth, optimal regulation of blood pressure and oxygenation. The authors also analyzed the direct resveratrol effects on the fetal heart in chronically catheterized pregnant sheep showing that resveratrol induced a significant increase in arterial blood oxygen saturation (SaO2), and fetal arterial oxygen partial pressure (PAO2). Parallelly, resveratrol supplementation did not affect SIRT1 mRNA levels or the activity of the AKT/mTOR or CAMKII signaling pathway in this model [206]. The authors concluded that maternal resveratrol supplementation could increase oxygenation and fetal growth in this animal model that mimics the fetal cardiac development conditions in humans and propose resveratrol as a possible candidate to restore fetal substrate supply in pregnancies with placental insufficiency [206]. Subsequently, the same authors reported the ability of resveratrol to increase body volume and fetal weight without affecting brain weight and volume and to optimize uterine artery blood flow, umbilical vein oxygen saturation, and fetal oxygen release. The authors concluded that increasing fetal weight might be beneficial to ameliorate fetal growth and oxygen supply impairment during pregnancy [207]. Employing a rat model, Bourque et al. [28] demonstrated that resveratrol could improve fetal outcomes associated with prenatal hypoxia, a common complication in pregnancy [28]. Indeed, resveratrol maternal intake during the gestational period (GD7 - GD21) was able to almost completely nullify fetal death in hypoxic pregnancies [28].

Another positive effect of resveratrol on fetal outcomes is its ability to increase fetal hemoglobin levels (HbF) [208]. Among the hemoglobinopathies, beta-thalassemia is characterized by a reduced synthesis of the beta-globin chain, with an unbalanced production of the globin chain, ineffective erythropoiesis, and anemia. This imbalance can be reduced by inducing an increase in gamma-globin gene expression, which combines with excess alpha-globin chains and produces HbF. Other events related to this disease lead to secondary tissue damage due to excess ROS production. [208]. In a study performed in human K562 cell line and erythroid precursors derived from normal donors and patients with beta-thalassemia, Fibach et al. [209] demonstrated that resveratrol (0–100 µM) exhibited antioxidant activity and stimulated the expression of gamma-globin genes leading to HbF accumulation [209]. A later study by Theodorou et al. [210] reported that resveratrol and one of its semi-synthetic derivatives act as (0–100 µM) therapeutic molecules carrying out an antioxidant effect and an inductive effect on HbF synthesis in primary erythroid progenitor cells from healthy donors. It is worth noting that the association of resveratrol and the drug decitabine caused a significant increase in hemoglobin induction activity above the level induced by the stilbene alone [210].

Moreover, a recent work by Bosquesi et al. [211] reported that both resveratrol and its synthetic derivatives (100 µmol/Kg) possess a potential positive effect in the treatment of sickle cell anemia (SCD) symptoms via the induction of gamma-globin. In CD34+ stem cells, resveratrol and one of its semi-synthetic compounds were able to induce twice the production of gamma-globin chains (γG + γA), compared to the vehicle. This semi-synthetic compound emerges as a new molecule in the gamma-globin inducers group. Aside from this property, anti-inflammatory and analgesic activities were also proven for these compounds suggesting them as an alternative in the treatment of SCD symptoms [211].

Resveratrol owns multiple positive health effects on different metabolic parameters, but it also possesses many different negative activities on the cellular life of various tissues/organs in different animal species [212,213,214,215]. In this context, the study [216] evaluated resveratrol’s effects on human fetal adrenocortical steroidogenesis during the 9–12th gestational weeks in vitro using human primary culture of fetal adrenocortical cells (HFAC) from glands of aborted fetuses. The authors showed that resveratrol (10 µM/24 h) significantly suppressed the synthesis of dehydroepiandrosterone, androstenedione, and 11- deoxycortisol in cells stimulated with adrenocorticotropic hormone (ACTH), an event associated with the inhibition of the activities and expression of cytochromes 17α-hydroxylase/17,20 lyase (CYP17) and 21-hydroxylase (CYP21). Based on the data obtained in this model, the authors advise against resveratrol intake by women who are in the early stages of pregnancy.

## 6. Conclusions and Future Directions

Resveratrol has been reported to possess numerous benefits against complicated pregnancies, with its activity elicited by regulating different physiological and cellular functions and modulating multiple intracellular signaling pathways implicated in pregnancy and pregnancy-associated conditions. Resveratrol demonstrated potent antioxidant activities by blocking DNA damage, regulating antioxidant enzymes, and modulating redox-regulated intracellular signals. Resveratrol also showed anti-inflammatory properties by inhibiting pro-inflammatory signaling pathways and the secretions of pro-inflammatory cytokines and growth factors. Resveratrol has the capability to cross the placenta, improve its oxidative status and directly affect the fetus during the gestational period producing beneficial effects for the reproductive performance and the offspring’s well-being. Resveratrol may offer potential therapeutic benefits for PE due to its antioxidative stress/anti-inflammatory properties that prevent cells from undergoing apoptosis and mediates a protective effect by decreasing blood pressure and attenuating vascular endothelial injury. Interestingly, resveratrol mediates a positive response in blood glucose regulation and attenuates GDM-related complications.

Despite the multiple reported benefits, the use of resveratrol in pregnancy needs to be better investigated to dissolve the high-dose-related toxicity issues highlighted by some papers and the intricate information derived from the available literature analysis. Indeed, although clinical trials indicate resveratrol safety for human consumption, results from the same studies are inconclusive regarding resveratrol’s protective effects against diseases and their complications. In particular, due to the vast difference in experimental models employed, duration of treatments, dosages, routes of administration, and formulations, the final interpretation of resveratrol effects on pregnancy and associated complications remains to be extrapolated.

Particular attention should be directed to performing systematic long-term clinical trials on pregnant women using determined resveratrol dosages to possibly reinforce and ultimately demonstrate resveratrol’s protective and therapeutic role. Specifically, the optimal resveratrol dosages to be employed in humans remains to be clarified. Indeed, ranges of biologically active resveratrol concentrations in vitro and in vivo experimental models differ enormously; hence, based on the currently available data, it is difficult to extrapolate the resveratrol concentration/s that provide the maximum beneficial effect in human subjects without provoking toxicity. This aspect is even more critical considering the resveratrol hormetic properties that tightly associate its final biological outcomes to the employed concentrations. Not to mention the interaction of resveratrol with the cellular and body redox state, which also influences its final effect in being beneficial or harmful. Furthermore, due to its poor bioavailability, as performed for other natural compounds [217,218,219], new resveratrol formulations that provide better molecule absorption and pharmacodynamics need to be developed and commercialized. In this light, many of the controversial resveratrol results in the literature might be due to the aforementioned aspects, which warrant more profound and systematic investigations to reach a final word on resveratrol therapeutic potentials.

**Figure 2:**
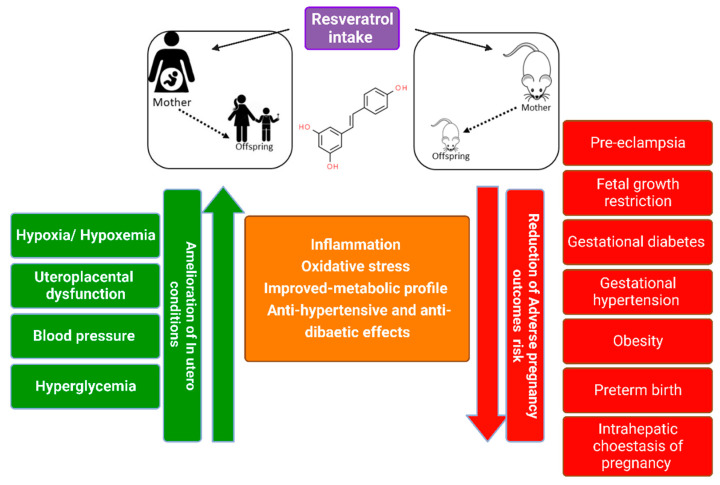
Maternal resveratrol supplementation decreases the risk of adverse pregnancy outcomes by ameliorating the in-utero conditions. Schematic representation of the resveratrol-impacted crosstalk between abnormal in-utero conditions and adverse pregnancy risk.

Overall, the data presented in our review indicates that resveratrol pregnancy consumption has numerous benefits on metabolic health for pregnant women and their offspring therapeutic approach in pregnancy-related pathological conditions such as elevated inflammation, preeclampsia, and GDM (Figure 2). Therefore, although some adverse effects have been highlighted at high dosages, clinical trials proved resveratrol safe for human consumption, making its recommendation by physicians to pregnant women conceivable. If that ever happens, the final validation of resveratrol therapeutic employment in pregnancy by evidence-based research would have massive consequences both on the mother and the fetus, allowing for early intervention and ensuring healthy mothers and children.

## Figures and Tables

**Table 1:** Studies using resveratrol in the treatment of models of pre-eclampsia in pregnancy.

Reference	Type of the Study	PE Experimental Model	Dose	Mechanism of Action	Outcomes of Supplementation in the Experimental Model
[94]	In vitro/Clinical trial	-ECs -Human	200 mL of polyphenol rich-grape fruit/1 µM of trans-resveratrol	NO, HO-1, miRNA expression, GSH, ARE, ROS levels	↑ ARE, NO, HO-1, GSH, ARE ↓ ROS, oxidative stress No studied effect in offspring
[96]	In vitro/Clinical trial	-ECs -Human	200 mL of polyphenol rich-grape fruit/1µM of trans-resveratrol	SIRT1 expression	No significant effect No studied effect in offspring
[102]	In vitro/In vivo	-HTR-8/SVeno cell culture/ -NG-nitro-l-arginine methyl ester mice model (L-NAME)	100 µmol/L resveratrol 20 mg/kg/day	Expression of genes regulating migration, invasion, angiogenesis and EMT-related factors in trophoblasts Hypertension and proteinuria measurement. Endothelial dysfunction/ injury	↑ E-cadherin, β-catenin, N-cadherin, and vimentin expression, ↓ Alteration of WNT-related gene expression, including WNT1, WNT3 and WNT5B. ↓ Hypertension and proteinuria No studied effect in offspring
[108]	In vitro	primary trophoblasts and HUVECs	0–100 μM	-Angiogenesis activation (sFlt-1) (sEng) secretion Anti-inflammatory effect -NFκB , IL-6 and IL-1β -Antioxidant effect HO-1, NQO1, GCLC, (TXN) Endothelial dysfunction, VCAM Endothelin-1 eNOS	↓ sFlt-1, sEng ↓ IL-6, IL-1β and TNF-α.‘ ↑, NQO1, GCLC TXN ↓ HO-1 protein in trophoblast. ↑ VCAM-1 ↓ Endotelin-1, ↑eNOS No studied effect in offspring
[109]	In vivo	NG-nitro-l-arginine methyl ester mice model (L-NAME)	20 mg/kg/day	-Antioxidant effect, Apoptosis SOD, MDA	↑ SOD, MDA No studied effect in offspring

Ecs: Endothelial cells, NO: Nitric oxide, HO-1: Heme oxygenase-1, miRNA: microRNA, GSH: Gluthatione, ARE: antioxidant response element, ROS: Reactive oxygen species, SIRT1: sirtuin1, WNT: WNT genes, sFlt-1: soluble fms-like tyrosine kinase-1, sEng: soluble endoglin, NFκB: nuclear factor-kappa B, IL-6: Interleukin-6, IL-1β: Interleukin-1β, TXN: thioredoxin, NADPH, NQO1: NADPH Quinone Dehydrogenase 1, TNF-α: Tumor necrosis factor-alpha, GCLC: Glutamate-Cysteine Ligase Catalytic Subunit, VCAM-1: Vascular cell adhesion molecule-1, eNOS: endothelial nitric oxide synthase, SOD: Superoxide dismutase, MDA: malondialdehyde.

**Table 2:** Studies using resveratrol in the treatment of models of gestational diabetes miletus (GDM) in pregnancy.

Reference	Type of the Study	GDM Experimental Model	Dose	Mechanism of Action	Outcomes of Supplementation in the Experimental Model
[116]	In vivo	Streptozotocin GDM pregnant rats model	120 and 240 mg/kg	Amelioration of blood glucose and blood lipids levels	↑ Insulin levels, ↓ blood glucose levels Amelioration of lipidic profile No studied effect in offspring
[117]	In vivo	Streptozotocin GDM pregnant rats model	500 μg/mL (CS-ZnO-RS)	Anti-diabetic effect Anti-inflammatory effect	↓ blood glucose levels lipid content reduced the level of ↓ IL-6 and MCP-1, GRP78, p-IRE1α, p-eIF2α, and p-PERK Inhibition of α-glucosidase and α-amylase No studied effect in offspring
[22]	In vivo	Streptozotocin GDM pregnant rats model	120 and 240 mg/kg	Amelioration of the lipids metabolic profile	↑ HDL-C and adiponectin ↓ leptin, resistin, TNF-α, IL-6 levels, the body weight, TC, TG, and LDL-C No studied effect in offspring
[142]	In vivo	high-fat fed hamster model	0.025%	Amelioration of the lipids metabolic profile	-HMG-CoA reductase expression No studied effect in offspring
[128]	In vivo	high-fat diet GDM mice model IR adipocyte model was established by dexamethasone-inducing	0.2%	Amelioration of glucose and lipids metabolic profile Amelioration of IR in adipocytes	↓ The bodyweight, serum glucose ↑ serum insulin Upregulations of miR-23a-3p/NOV axis ↑ Adiponectin, Leptin, p-PI3K, and p-Akt No studied effect in offspring
[118]	In vivo	female Sprague-Dawley rat model, fed with a high-fat and sucrose diet	147 mg/kg/day	Protection against gestational diabetes-induced glucose intolerance and islet dysfunction	-Restored glucose tolerance, normoglycaemia and improved insulin secretion in offspring -Attenuation of hepatic steatosis, insulin resistance, glucose intolerance and dysregulated gluconeogenesis in offspring -Downregulation of lpl, ppara, g6p genes
[144]	In vivo	C57BL/KsJ-Lep (db/+) (db/+) genetic GDM pregnant mouse model	10 mg/kg/day	Amelioration of glucose metabolic profile via SIRT1/AMPK pathway Amelioration of IR	↑ Glucose metabolism, insulin tolerance and reproductive outcome of the pregnant db/+ females AMPK activation ↓ glucose-6-phosphatase in both pregnant db/+ females and their offspring

GDM: Gestational diabetes miletus, CS-ZnO-RS: resveratrol-zinc oxide complex encapsulated with chitosan, IL-6: Interleukin-6, MCP-1: Monocyte Chemoattractant Protein-1, GRP78, p-IRE1α, p-eIF2α, and p-PERK: endoplasmic reticulum stress components, TNF-α: Tumor necrosis factor-alpha, TC: Total cholesterol, TG: Triglycerides, LDL-C: low-density lipoprotein-C, IR: Insulin resistance, miR-23a-3p/NOV axis: microRNA-23a-3p, p-Akt: phosphorylated-AKT, p-PI3K: Phosphoinositide 3-kinase, lpl: lipoprotein lipase gene, ppara: Peroxisome proliferator-activated receptor alpha, g6p: glucose-6 phosphatase. AMPK: AMP-activated protein kinase.

## Data Availability

Not applicable.
